# Computational and Statistical Approaches for Modeling of Proteomic and Genomic Networks

**DOI:** 10.1155/2013/561968

**Published:** 2013-05-16

**Authors:** Mohamed Nounou, Hazem Nounou, Erchin Serpedin, Aniruddha Datta, Yufei Huang

**Affiliations:** ^1^Chemical Engineering Program, Texas A&M University at Qatar, Doha, Qatar; ^2^Electrical and Computer Engineering Program, Texas A&M University at Qatar, Doha, Qatar; ^3^Electrical and Computer Engineering Department, Texas A&M University, College Station, TX 77843, USA; ^4^Electrical and Computer Engineering Department, University of Texas at San Antonio, San Antonio, TX 78255, USA

Understanding and characterizing the behavior of biological systems triggered a huge interest among researchers to better understand how genes and proteins interact within cells by developing complex networks of structural, metabolic, and regulatory pathways. Advances in sensing technologies allowed collecting high throughput genomic and proteomic data that can be used in inferring the structure of proteomic and genomic networks. However, developing reliable algorithms for inferring genomic and proteomic networks and developing intervention techniques that can modify the behavior of biological systems are hindered by several factors. The most stringent limitations are the underdetermined nature of available data, which manifests in the large number of unknown variables and the small number of data samples, and the stochastic nature of the regulatory networks, which are often corrupted by noise and unknown latent variables during measurement. Therefore, developing computationally efficient data fusion, modeling, and intervention algorithms to overcome these limitations represents currently one of the most important research challenges in the field of computational biology. In this special issue, the authors have developed statistical and control techniques to model biological systems and to design intervention strategies that can help better understand these systems and can lead them to their more desirable states. This special issue consists of seven papers that address research topics along the lines mentioned previously. More details about the contributions of each paper in this special issue are presented next.

The paper “*Gene regulation, modulation, and their applications in gene expression data analysis*” by M. Flores et al. provided a unified mathematical description of the modulation of gene regulation, encompassing earlier mRNA expression-based methods and the more recent ceRNA method. The paper also presented applications to illustrate the construction of regulation networks, modulation effects, and the preliminary findings from these networks.

The paper “*Spectral analysis on time-course expression data: detecting periodic genes using a real-valued iterative adaptive approach*” by K. S. Agyepong et al. presented a novel scheme for detecting periodicities in time-course expression data using a real-valued iterative adaptive approach (RIAA), which is usually applied for periodogram estimation in signal processing. The spectrum obtained from spectral analysis is then analyzed using the Fisher's hypothesis test, and using a proper threshold, periodic genes can be detected. The detection scheme is illustrated through its application to simulated and real datasets. 

The paper “*Identification of robust pathway markers for cancer through rank-based pathway activity inference*” by N. Khunlertgit and B.-J. Yoon presented an enhanced pathway activity inference method that uses gene ranking to predict the pathway activity in a probabilistic manner. This inference method is used to identify robust pathway markers that can ultimately lead to robust classifiers with reproducible performance across different genetic datasets. The advantages of the proposed method are illustrated through its application to breast cancer data. 

The paper “*An overview of the statistical methods used for inferring gene regulatory networks and protein-protein interaction networks*” by A. Noor et al. provided a review of the most important statistical methods used for modeling gene regulatory networks (GRNs) and protein-protein interaction (PPI) networks. The paper focused on the recent advances in the statistical graphical modeling techniques, state-space representation models, and information theoretic methods that were proposed for inferring the topology of GRNs.

The paper “*MRMPath and MRMutation, facilitating discovery of mass transitions for proteotypic peptides in biological pathways using a bioinformatics approach*” by C. Crasto et al. described two software packages called MRMPath and MRMutation that are used to extract information from genomic data related to quantitative mass spectrometry analysis, such as the mass-to-charge ratio (m/z) values of proteotypic peptides and product ions. MRMPath utilizes publicly available information related to biological pathways from the Kyoto Encyclopedia of Genes and Genomes (KEGG) database. MRMutation, on the other hand, catalogs and makes available, following processing, known (mutant) variants of proteins from the current UniProtKB database. 

The paper “*Intervention in biological phenomena via feedback linearization*” by M. A. Fnaiech et al. presented an intervention technique to move biological systems from an undesirable state to a more desirable state. The authors considered biological phenomena represented by S-systems, and designed an intervention technique based on feedback linearization of the system model. The developed intervention technique is illustrated through its application to the glycolytic-glycogenolytic pathway model. 

The paper “*Reverse engineering sparse gene regulatory networks using cubature Kalman filter and compressed sensing*” by A. Noor et al. presented a novel algorithm for inferring gene regulatory networks from time-series data. The algorithm makes use of the cubature Kalman filter (CKF) and the Kalman filter (KF) techniques in conjunction with compressed sensing methods to model the gene network as a state-space model. The developed algorithm is evaluated using simulated as well as real biological data sets, which include the DREAM4 *in silico* data sets and the *in vivo* data sets from the IRMA network.

*Mohamed Nounou* *Hazem Nounou* *Erchin Serpedin* *Aniruddha Datta* *Yufei Huang*

